# Atypical Presentation of Pediatric Spinal Tuberculosis as Chronic Abdominal Pain: A Case Report

**DOI:** 10.1002/ccr3.72125

**Published:** 2026-02-23

**Authors:** Bilal Aslam, Faiza Farooq, Shafiq Ur Rahman, Fazeela Bibi, Umama Alam, Okasha Tahir, Adil Nawaz, Jibran Ikram

**Affiliations:** ^1^ University of Lahore Lahore Punjab Pakistan; ^2^ Hod Radiology, University of Lahore Teaching Hospital UCMD Lahore Punjab Pakistan; ^3^ Saidu Group of Teaching Hospital Swat Pakistan; ^4^ Jinnah Medical and Dental College Karachi Sindh Pakistan; ^5^ Khyber Medical College Peshawar Khyber Pakhtunkhwa Pakistan; ^6^ Bacha Khan Medical College Mardan Khyber Pakhtunkhwa Pakistan; ^7^ Cleveland Clinic Foundation Cleveland Ohio USA

**Keywords:** anti‐tuberculous therapy, childhood TB, dorsal spine, kyphosis, pott's disease, spinal tuberculosis

## Abstract

Spinal tuberculosis, also known as Pott's disease, in children is a rare manifestation of extrapulmonary tuberculosis. The nonspecific clinical features, such as chronic abdominal pain, of extrapulmonary TB or spinal TB in children tend to delay diagnosis. We present the case of a 14‐year‐old girl who has been suffering from abdominal and backpain associated with spinal tuberculosis. This is a case study reporting on the treatment and outcome of a 14‐year‐old girl who presented with progressive lower abdominal and back pains for 6 months, accompanied by slight kyphotic deformity. The involvement in this case was multilevel dorsal vertebral destruction from D5 to D12, and both paravertebral and right psoas abscesses on the magnetic resonance imaging and thin‐section computed tomography scans were very suggestive of spinal tuberculosis. A biopsy during the operations was not done; the treatment strategy and the plan were based on the diagnosis made from the clinical presentation and imaging studies. The justification for not doing the biopsy when the clinical features and imaging became concordant with the diagnosis of spinal tuberculosis would mean giving less importance to the biopsy result. Clinical and radiological improvement was good, with some improvement in the angle from 38° to 22° and VAS score for pain from 8/10 to 2/10 after 6 months. The treatment plan consisted of posterior surgical decompression and stabilization with anti‐tubercular therapy for 12 months. This particular case underscores the fact that tuberculosis of the spine can have a presenting symptom of chronic abdominal pain in children. Radiographic assessment plays a pivotal role when histologic confirmation is impossible initially, and prompt multidisciplinary care, including radiology and anti‐tuberculosis treatment, prevents catastrophic consequences.

**Key Clinical Message**

- Spinal tuberculosis may very rarely manifest in children with unusual symptoms such as abdominal pain, causing delay in diagnosis and loss of spinal correction in children. Physicians in areas with spinal tuberculosis epidemics need to have a high index of suspicion for making the diagnosis of spinal tuberculosis in children with no discernible explanation for their back or abdominal pain. Radiographic findings need to guide the diagnosis when the diagnosis cannot be made with microbiological studies, and the diagnosis needs to not be delayed if it fits the disease.

## 1 Introduction

Spinal tuberculosis or “Pott's disease” remains a major challenge for public health, especially within endemic areas. Spinal TB still remains a common cause of spinal deformities and neurological injury among children despite advancements in technology and management approaches for its diagnosis and treatment [1]. Spinal TB within children can be distinguished from spinal TB within adults, in that spinal TB within children typically advances more rapidly in terms of bone vascularity [2]. Spinal TB often presents with vague symptoms, like backpain, weakness, and low‐grade fever, in children, causing a delay in treatment [3]. With progression, there may be collapse, paraspinal abscess, or cord compression, causing profound neurological impairment, like paraplegia, due to nerve damage [4]. The imaging modality of choice, based on modern technology, has shifted toward MRI, allowing visualization of specific features like destruction of vertebrae, soft tissue, or epidural abscess [5]. It is important to note, however, that established diagnoses depend on microbiological or histopathological evidence, underlining the importance of biopsy or PCR techniques for MTB isolation [6].

## 2 Case Presentation

In Lahore, a 14‐year‐old female class 6 student was referred to us with symptoms of progressive pain in the left upper quadrant and back for six months. Pain was insidious in onset and progressive in nature with exacerbations of pain with sitting and resolution with lying down. Along with pain, there was substantial unexplained weight loss. Although numerous visits were made at various clinics, there was only NSAID‐induced pain control with no other evaluation. There was no known exposure to tuberculosis, and she was from a lower–middle‐class family.

At first, her spinal examination results were ignored by the initial doctors, perhaps because her abdominal tenderness was more apparent. This further led to a delay in her diagnosis. Once referred, her spinal examination revealed tenderness in the thoracolumbar region and mild kyphosis. Clinical examination found the patient to be thin and malnourished. Her vital signs remained normal. Also, on spinal examination, there was tenderness, mild kyphosis, and restriction in the range of motion, most pronounced on forward bending. But there was no abnormality in the neurological examination. Other system examinations remained normal.

Informed consent and parental assent were obtained for treatment as well as for publishing these data, including anonymized radiographic images.

## 3 Investigations and Differential Diagnosis

**FIGURE 1:**
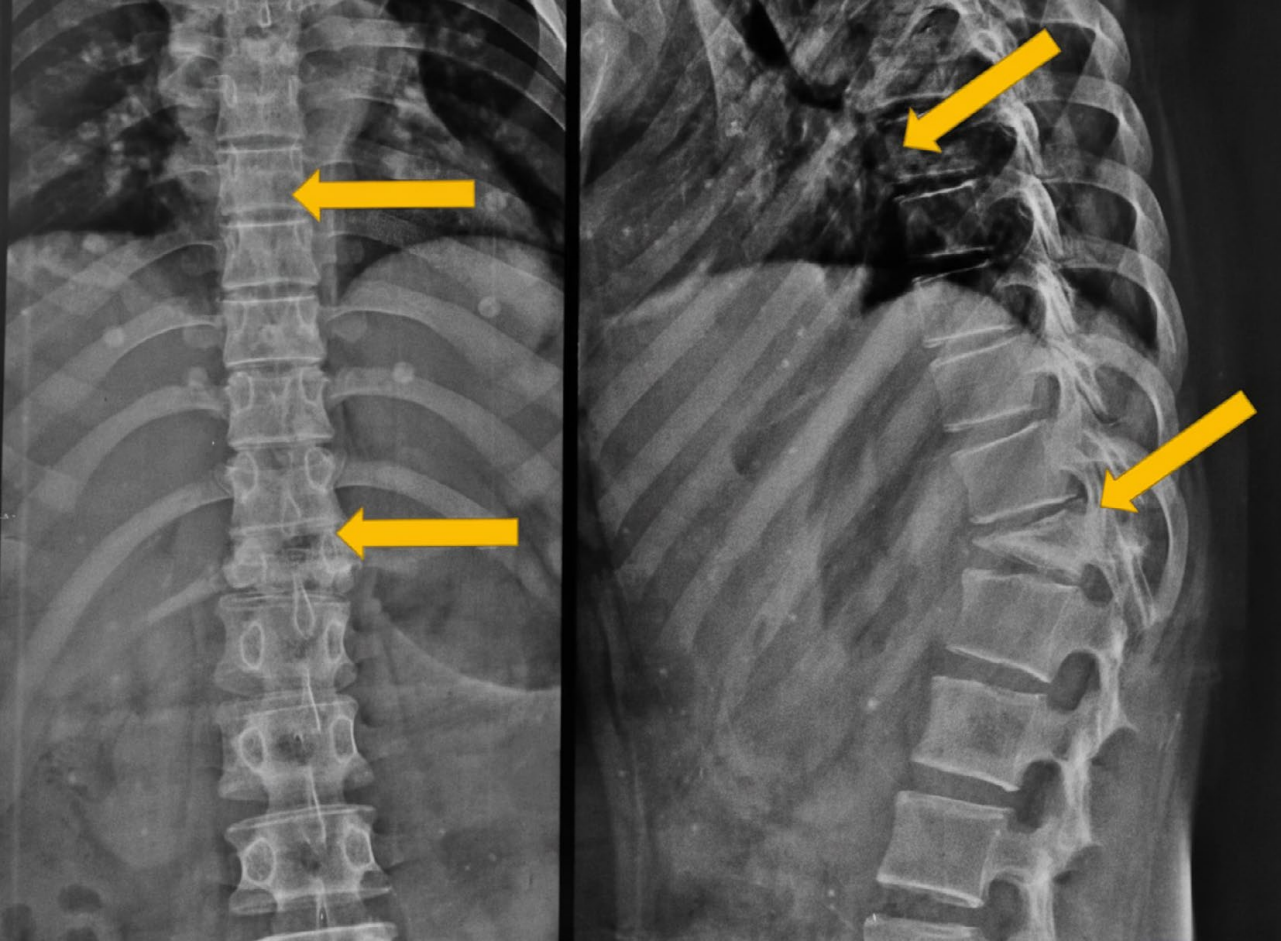
Plain radiographs of the dorsolumbar spine in anteroposterior and lateral views demonstrating vertebral body destruction and loss of vertebral height, suggestive of an infective pathology.

Initial lab work revealed mild anemia of 11 g and elevated ESR. Abdominal ultrasound revealed an unremarkable scan. No abnormalities were detected in the liver, pancreas, spleen, or kidneys, effectively excluding an intra‐abdominal process for her left‐upper quadrant abdominal pain. The spinal MRI not only looked for pathology within her spine but also adjacent abdominal structures, which were unremarkable, again effectively excluding an underlying abdominal process. Antero‐posterior and lateral radiographs of the dorsolumbar spine (Figure 1) revealed destruction of the vertebral bodies, consistent with an infectious process like tuberculosis, eosinophilic granuloma, or a pyogenic infection. The scans indicated the need for an MRI study, but the patient was initially lost to follow‐up.

**FIGURE 2:**
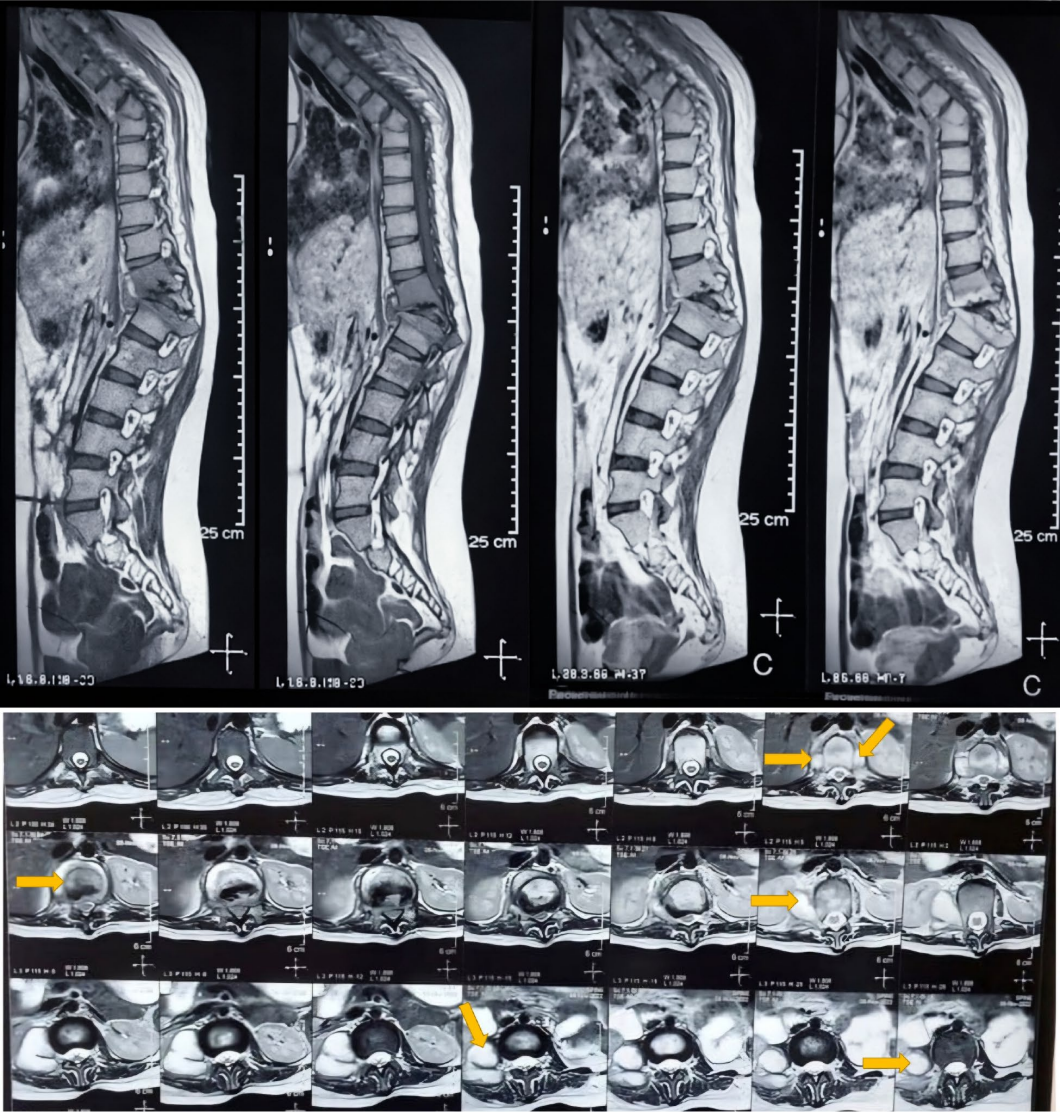
Magnetic resonance imaging (MRI) of the spine showing vertebral body destruction with adjacent soft‐tissue involvement, consistent with tuberculous spondylitis.

She came back to the OPD after several months with an MRI report that confirmed tuberculous spondylitis, which had destroyed vertebral bodies and had involvement of the surrounding soft tissue (Figure 2). Inflammation markers were raised, and LFT and RFT were within normal limits. The viral serologies were negative, and urine examination was unremarkable.

**FIGURE 3:**
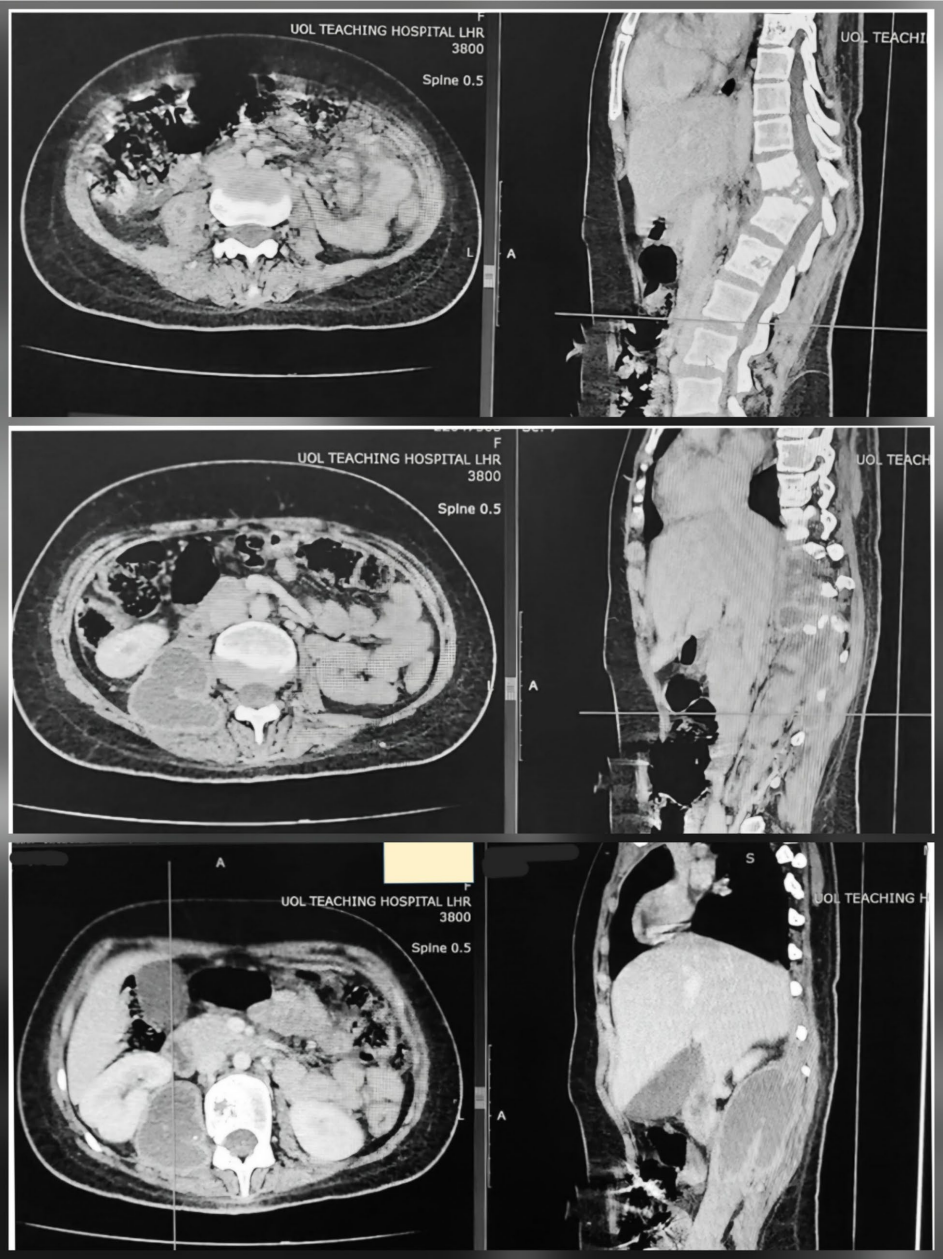
Contrast‐enhanced computed tomography (CECT) of the abdomen revealing extensive destruction of multiple dorsal vertebral bodies with associated paravertebral and right psoas abscess formation, highly suggestive of Pott's disease.

CT scan revealed complete destruction of DV5 and DV12 vertebral bodies with contiguous vertebral involvement and paravertebral as well as right psoas abscesses (Figure 3), highly suggestive of Pott's disease. No intraoperative biopsy was taken; the diagnosis was made primarily on clinical and radiological grounds after team consensus. The rationale for omitting biopsy was that both clinical presentation and radiological findings were strongly in support of spinal TB, and waiting for histopathological confirmation could have led to further vertebral collapse and worsening deformity.

**FIGURE 4:**
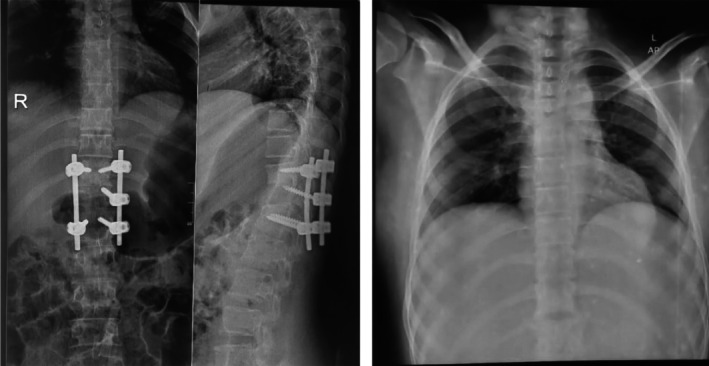
Postoperative radiograph of the chest and spine demonstrating adequate spinal decompression and alignment following surgical intervention.

Owing to the severe spinal damage and presence of kyphotic deformation, the patient was taken up for surgical decompression and spinal stabilization. The strategy adopted was posterior only surgery on the worst areas to relieve the spinal cord compression and stabilize the spinal areas affected by the TB infection and subsequent damage. A posterior‐only approach was selected, considering the patient's age, the limited availability of instrumentation for anterior support, and to minimize morbidity. Anterior column support was not employed since the posterior construct was able to achieve adequate stability. Adequate decompression and alignment were confirmed on post‐operative imaging (Figure 4).

## 4 Outcome and Follow‐Up

The patient was started on a standard treatment regimen for tuberculosis, which consisted of a 2‐month intensive phase of isoniazid, rifampin, pyrazinamide, and ethambutol and a 10‐month continuation phase of isoniazid and rifampin. The patient received supportive treatment in the form of NSAIDs for pain relief, vitamin supplements, and physiotherapy.

At follow‐up, the patient demonstrated significant improvement, including relief of pain and increased mobility of the spine. Pain scores were reduced from 8/10 preoperatively to 2/10 postoperatively, using the VAS scale, and the kyphosis angle reduced from 38° to 22°. The inflammatory markers were slowly reducing, and follow‐up imaging would be undertaken to observe the changes in the spine. At the 6‐month follow‐up, the child had recovered spine strength and could walk independently. This study has been reported according to the SCARE guidelines [7].

## 5 Discussion

Spinal tuberculosis, also known as Pott's disease, is the most frequent manifestation of skeletal tuberculosis, contributing to about 50% of all skeletal tuberculosis cases. It mostly occurs in the thoracic and lumbar regions. It can result in destruction, deformities, and neurological complications. Spinal tuberculosis, despite being a disease with typical backpain and neurological symptoms, can often have extrapulmonary manifestations such as chronic abdominal pain, which can result in delayed diagnosis and treatment [1, 8]. Visceral pain in the abdominal region due to the primary manifestation of TB of the spine is rare but a notable finding. This pain could be due to irritation of the sympathetic plexus, psoas muscle involvement, or compression of the adjacent structures. The lower thoracic and upper lumbar areas are close to the celiac plexus and mesenteric nerves, leading to referred abdominal pain, due to the potential for abdominal or renal abnormalities [8, 9, 10]. Moreover, psoas abscesses, a common consequence of spinal TB, could spread to the retroperitoneal region, giving rise to tenderness, fever, or mimicking the diagnosis of appendicitis or abdominal abscesses [9, 11]. In our case, the LP quadrant pain was initially confusing, but careful work‐up did not include abdominal causes of the pain, including spleen, pancreas, kidney, and liver disease, by ultrasound and MRI correlation, and therefore solidified the fact that the pain was only referred to and came from the spine.

Early and correct diagnosis of spinal TB has a great preventive role in terms of serious complications like paralysis. MRI has been considered the imaging modality of choice because it shows bone marrow edema, bony destruction, paravertebral abscesses, and spinal cord compression before the appearance of significant bony destruction [11, 12]. CT scan assesses the degree of bone destruction and kyphosis; plain radiographs can also be used in the late stages. MRI and CT scan results in the patient were highly suggestive of spinal TB and enabled the early treatment of the patient despite the lack of confirmatory histopathology. The mainstay of treatment for spinal TB is ATT for 9 –12 months, with an initial intensive phase followed by a continuation phase [4]. Surgical intervention is indicated for cases with instability of the spine, progressive neurological deficits, or large abscess formation [13]. In our case, surgery was considered for the progressive deformity, extensive destruction of the vertebrae, and impending neurological compromise. Follow‐up after the treatment is necessary to observe the regression of the disease process and to avoid deformities [14]. The objectives of surgery are to decompress the nervous elements, correct deformity, and establish stabilization. For pediatric cases, posterior‐only instrumentation is now gaining popularity because of lesser morbidity, reduced blood loss, and sufficient correction generated for flexible spines [2, 3]. This particular case needed rapid decompression without the dangers associated with the anterior approach. Our case adds to the literature that abdominal pain can be one of the misleading presentations of pediatric spinal tuberculosis, which may delay diagnosis. There have been a few similar reports [10, 15] presenting children whose only chief complaints were symptoms of the abdomen, so the authors suggested that the clinicians should always keep in mind spinal causes for the complaints, even if the abdominal imaging was unremarkable. We document this case, including radiological and clinical correlation, to add to early recognition and management strategies for atypical manifestations of spinal TB.

## 6 Conclusion

This case highlights the value of prompt attention to chronic back or abdominal pain in children and the value of considering the possibility of TB in patients in regions where the disease is common. The treatment was not delayed pending a biopsy because biopsy could have made treatment more difficult or impossible. The delay in diagnosis resulted in substantial damage to the spine, hence the need for surgery. Our case further emphasizes the value of commencing treatment when the clinical picture, in association with the radiographic results, is typical, especially when a biopsy is pending. It is vital to consider the value of referral from family physicians to avoid such undesirable outcomes in patients with spinal TB.

## Author Contributions

**Bilal Aslam:** conceptualization, validation, writing – original draft. **Faiza Farooq:** validation, writing – original draft. **Shafiq Ur Rahman:** visualization, writing – original draft. **Fazeela Bibi:** writing – original draft, writing – review and editing. **Umama Alam:** validation, writing – review and editing. **Okasha Tahir:** data curation, visualization, writing – review and editing. **Adil Nawaz:** resources, writing – review and editing. **Jibran Ikram:** data curation, resources, visualization.

## Funding

The authors have nothing to report.

## Ethics Statement

The publication of this case report has been authorized by the quality service of our institution as case reports are exempted from ethical approval in our institute.

## Consent

Written informed consent was obtained from the patient's parents/legal guardian for publication and any accompanying images. A copy of the written consent is available for review by the Editor‐in‐Chief of this journal on request.

## Conflicts of Interest

The authors declare no conflicts of interest.

## Data Availability

Due to privacy and ethical restrictions, the data supporting the findings of this study are available from the corresponding author upon reasonable request.
